# Surgical capacity in ophthalmology: the unmet need for sustainable solutions

**DOI:** 10.1038/s41433-025-04153-x

**Published:** 2025-12-16

**Authors:** Roxane J. Hillier, Andrew Chang, Amanda Matse-Orere, Christian Bindesbøll, Larissa S. Moniz, Victoria Heaton, John Petrie, Callum Bannister

**Affiliations:** 1 Newcastle Eye Centre, Royal Victoria Infirmary, Newcastle-Upon-Tyne, UK; 2 Sydney Retina Clinic & Day Surgery, Sydney, NSW Australia; 3 Sydney Eye Hospital, Sydney, NSW Australia; 4 F. Hoffmann-La Roche AG, Basel, Switzerland; 5 Fighting Blindness Canada, Toronto, ON Canada; 6 Macular Disease Foundation Australia, Sydney, NSW Australia; 7 Putnam Associates, Newcastle-Upon-Tyne, UK

**Keywords:** Health care, Health occupations

## Abstract

The increasing prevalence of eye diseases is placing significant pressure on surgical and procedure-based ophthalmology services worldwide. Delays in surgical care can lead to poorer patient outcomes and reduced treatment efficacy, highlighting the urgent need for healthcare systems to address these challenges. This narrative review provides a broad overview of surgical and procedure-based ophthalmology capacity constraints across five countries (UK, Germany, Australia, Singapore, and India) to identify cross-cutting, system-level challenges that transcend individual diseases or interventions, thereby informing policy and investment strategies. It examines contributing factors from patient, clinician, and healthcare system perspectives, focusing on workforce shortages, operating theatre limitations, and scheduling conflicts, while also addressing diagnostic and medical challenges affecting surgical pathways, preoperative preparation, and postoperative care. Key insights were derived from targeted literature searches and supplemented by qualitative expert interviews. The searches revealed themes including the rising prevalence of retinal diseases, workforce gaps, and the impact of capacity constraints on clinical outcomes. Expert interviews provided nuanced, qualitative perspectives from ophthalmic surgeons on local challenges and opportunities for improvement. Proven and prospective solutions were identified, including task shifting, technological innovations, and workflow optimisation. Examples such as AI-assisted diagnostics, mobile clinics, and telemedicine have successfully mitigated capacity constraints in various healthcare systems. By identifying actionable strategies, this review serves as a call-to-action to healthcare policy makers to improve surgical and procedure-based service capacity, enhance patient access to care, and ultimately optimise clinical outcomes.

## Introduction

The ever-growing prevalence of vision-threatening eye diseases [1–8], including diabetic macular oedema (DMO/DME), diabetic retinopathy (DR), cataracts, and retinal detachment, driven by an ageing population [9], is placing increasing pressure on surgical and procedure-based ophthalmology services worldwide [10]. In the UK alone, The Royal College of Ophthalmologists’ (RCOphth) 2022 census highlighted that surgical backlog was up 14% from 2019, due in part to the COVID-19 pandemic [11]. Despite advances in medical technology, the expanding patient pool has led to significant capacity constraints (CC) in ophthalmology clinics and hospitals. Delays in ophthalmic care can result in reduced treatment effectiveness and worse patient outcomes [12–15], therefore, addressing these delays is critical.

**Fig. 1:**
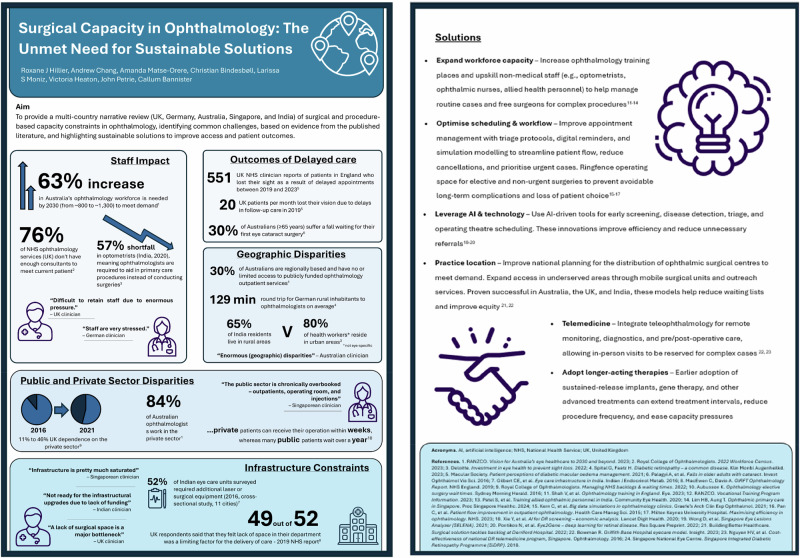
Overview of global surgical capacity constraints in ophthalmology and their impact on access to care. The infographic summarises key challenges in surgical and procedure-based capacity across multiple countries (UK, Germany, Australia, Singapore, and India), including workforce shortages, staff stress, outcomes of delayed care, geographic inequities in service availability, differences between public and private sector provision, and infrastructure limitations. Icons represent clinicians, patients, travel distance, and health-system settings used to illustrate the thematic areas.

Whilst increasing prevalence of eye diseases [1–6] and general CC in ophthalmology have been reported previously in literature [11, 16], very few studies have specifically explored ophthalmology CC in the context of surgical and procedure-based (e.g., intravitreal injection) ophthalmology. This review was deliberately designed to take a broad, multi-country perspective (UK, Germany, Australia, Singapore, and India), examining current and future CC (i.e., next 10 years) in surgical and procedural ophthalmology, focusing on retinal diseases and cataracts (the most common non-retinal surgical condition), across five healthcare systems. The healthcare systems were chosen for their geographic diversity, variations in economic development, and distinct healthcare systems encompassing both public and private sectors. By adopting this breadth, our aim is to highlight overarching, system-level challenges and cross-cutting themes that would not be captured by focusing narrowly on a single disease or intervention. Contributing factors are identified, focusing on the perspectives of patients, clinicians, and allied health workers, and how these factors impact patient outcomes and healthcare staff and services. In addition, both proven and prospective solutions are discussed. By doing so, we aim to deliver a comprehensive, evidence-based, clear call-to-action to policymakers that encourages sustainable investment in healthcare systems to improve efficiency and ensure more equitable access to surgical care in ophthalmology. A summary of the key findings are presented via infographic in Fig. 1.

## Methods

### Overview

Targeted literature searches were conducted to provide a comprehensive overview of CC and proposed solutions in surgical ophthalmology across 5 countries, chosen for their geographic diversity, variations in economic development, and distinct healthcare systems encompassing both public and private sectors: UK, Australia, Germany, Singapore and India. Expert interviews were conducted to supplement findings from the literature searches, providing insight from key opinion leaders (KOLs). This broad, multi-country approach was deliberate, designed to capture diverse system-level challenges and solutions rather than to focus narrowly on one condition or intervention type.

### Search strategy

The search strategy was designed to capture relevant publications and data sources related to ophthalmic surgical CC, including workforce limitations, infrastructure challenges, etc. Targeted searches were conducted in August 2023 in PubMed and Google Scholar using a combination of controlled vocabulary terms and free-text keywords, such as ‘capacity constraints’, ‘retinal disease’, ‘ophthalmology’, ‘referral’, ‘waiting’, etc. The search strategy was refined iteratively to balance sensitivity and specificity.

### Screening and data extraction

A full list of PICOS criteria (Population, Intervention, Comparator, Outcomes, Study design) is provided in the Supplementary Information (Supplementary Table 1). Titles and abstracts were screened for relevance by 2 reviewers independently. Full texts of potentially eligible studies were retrieved and assessed against the PICOS criteria. Any discrepancies in selection were resolved through discussion or consultation with a third reviewer. Data were extracted into a structured data extraction table, which included information on study design/type, sample size, geographic focus, disease focus, and outcomes related to CC.

### Analysis of targeted search outcomes

**Fig. 2:**
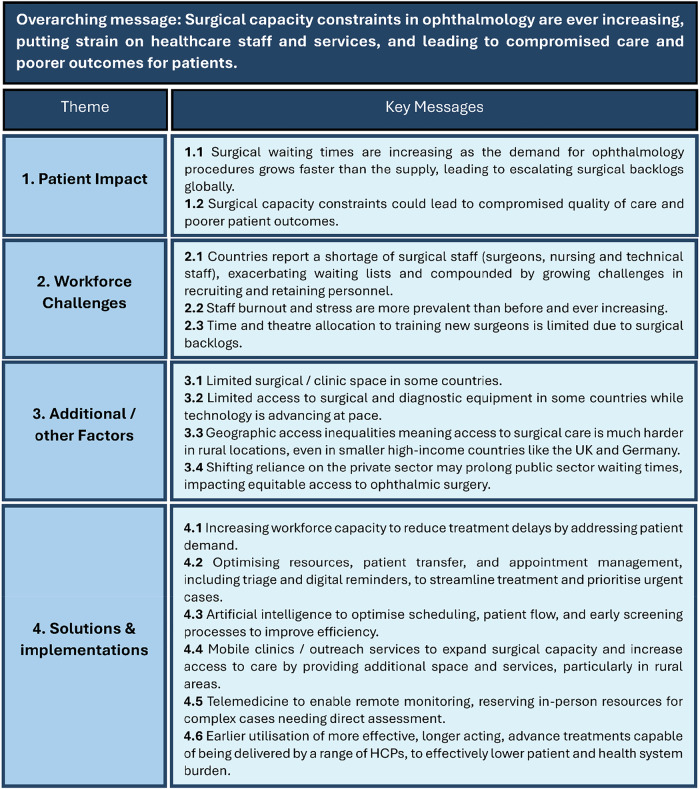
Surgical capacity constraints in ophthalmology across six countries. This figure summarises key themes identified in a multi-country narrative review (UK, Germany, Australia, Singapore, and India) on surgical and procedure-based capacity limitations in ophthalmology. Panel 1 highlights the patient impact of capacity constraints. Panel 2 illustrates workforce pressures, including projected workforce needs, current consultant shortages, optometrist shortfalls, and clinician-reported stress. Panel 3 presents additional factors including limited operating spaces, access to equipment and geographic disparities. Panel 4 suggests solutions such as expanding workforces and introducing AI.

Extracted data were synthesised narratively, focusing on prevalence of eye diseases, workforce, clinic space, equipment availability, waiting times/lists, appointment volume/demand, clinical impact, barriers, and solutions (Fig. 2).

### Expert interviews

Findings from the targeted searches informed the design of the expert interviews, ensuring alignment with both identified trends and knowledge gaps. Experts were identified through targeted searches of hospital websites and then screened for eligibility before invitation. The sampling frame consisted of ophthalmic surgeons meeting the following criteria:

- Active, consultant-level ophthalmic surgeons
- Minimum of 6 years’ experience in ophthalmic care
- Direct involvement in clinical management
- Experience in managing an ophthalmology department or clinic
- Practising in secondary or tertiary care in one of the five study countries

Ten clinicians were interviewed in total, with 2 from each country. Each completed a pre-interview questionnaire (1 h) and participated in a 30-min semi-structured interview, following a standardised interview guide (Supplementary Table 2). Clinicians were reimbursed for their time. Interviews were recorded, transcribed, and analysed together with the pre-interview questionnaires using thematic coding. Themes were initially informed by the literature review and refined as new issues emerged from the interviews. Across the ten interviews, the same core themes recurred, indicating that additional interviews were unlikely to generate substantially new insights. Findings are presented as qualitative insights and contextual quotes and are clearly labelled as such where used, not as statistically significant or generalisable data.

## Insights into the scale of demand

In addition to their geographic diversity, population size, variations in economic development, and distinct healthcare systems, the five countries also differ in terms of patient demographics, the scale of their ageing populations, and the prevalence of eye conditions. These variations provide valuable insights into how different healthcare infrastructures manage rising demands in eye care. By examining these diverse settings, we aim to highlight common and healthcare system-specific challenges, as well as identify opportunities for optimising ophthalmic care across various contexts.

In 2020, it was estimated that more than 5% of the total population in each healthcare system experienced some form of vision loss, except for India, where the prevalence was nearly 20%, Table 1. However, causes vary: in high-income countries, vision loss is primarily driven by age-related conditions such as age-related macular degeneration (AMD), DR, and glaucoma [1–4, 6], whereas in India, higher rates stem from uncorrected refractive error, corneal infections, and untreated cataracts, reflecting disparities in access to treatment [17]. Currently, the most common eye diseases requiring surgical or procedural intervention globally include cataracts, retinal detachment, glaucoma, and DR, but new treatment innovations of AMD and DME will add to the demand for surgical intervention. Cataracts and retinal detachment always require surgery, whereas glaucoma and DR are primarily managed procedurally through laser therapy or intravitreal injections, with surgical intervention reserved for severe or refractory cases.

**Table 1:** Economic classification (2022), vision loss and blindness estimates (2020), and ophthalmologists’ population by country.

Country	Economic classification (World Bank and IMF) [90, 91]	Estimated population with vision loss (per million, %) [82]	Estimated blind^a^ population (per million, %) [82]	Ophthalmologists per million^b^ [44]
Australia	High income; advanced economy	50,980 (5.3)	2235 (0.2)	29
Germany	High income; advanced economy	69,212 (6.9)	2745 (0.3)	81
India	Lower-middle income; emerging economy	195,652 (19.3)	6667 (0.6)	11
Singapore	High income; advanced economy	71,795 (7.1)	2222 (0.2)	24
United Kingdom	High income; advanced economy	63,328 (6.4)	2504 (0.3)	49

^a^Blind is defined as <3/60 or less than 10° visual field around central fixation [82].

^b^As values differ by source, for consistency, data were sourced from a single, reputable, global source: the International Council on Ophthalmology (ICOPH).

Ophthalmic surgeries can be classified as urgent or elective, depending on the severity of the condition and the need for timely intervention. Urgent surgeries require immediate action, often leading to the postponement of elective cases at short notice. The incidence of urgent surgeries, such as rhegmatogenous retinal detachment repair, is rising across the analysed healthcare systems, exacerbating CC [18–21]. Furthermore, with DR prevalence predicted to continue rising [4, 19, 22, 23], the demand for common, acute vitreoretinal surgeries, is also expected to rise. A nationwide database study of Australian public and private hospitals revealed that the total number of vitreoretinal procedures (including both urgent and elective surgeries) jumped from 8102 in 2001 to 136,430 in 2019 (1584% increase). The incidence of elective surgeries is also growing globally with prevalence expected to continue increasing, including cataracts [4, 22, 24, 25] and elective macular surgeries [20, 21].

### Patient impact

Surgical waiting times are increasing as the demand for ophthalmology procedures grows faster than the supply, leading to escalating surgical backlogs globally.

#### Ophthalmic surgical backlogs: a global challenge of rising demand and limited supply

The demand for ophthalmology services is growing globally. Over the last decade in the UK alone, outpatient appointment volume has increased by 40%, with a year-on-year increase in surgical activity [26]. Ophthalmologic care contributes the largest number of outpatient appointments in the NHS, exceeding 7.5 million (equivalent to ~110 per 1000 population) with over 500,000 surgical procedures (~7.4 per 1000 population) in 2023 [26]. According to the 2022 RCOphth UK Workforce Census, over 200,000 more patients were waiting to see an ophthalmologist in 2022 compared to 2018, equivalent to ~3 additional patients per 1000 population [11]. Unsurprisingly, surgical backlogs are commonly reported; compared with 12 month ago, 74% of eye units are more concerned about the impact of outpatient backlogs on patient care, with 63% estimating it will take at least a year to clear (26% estimated > 3 years) [11]. Backlogs were exacerbated by the COVID-19 pandemic where supply was severely reduced, a trend noticed across all investigated countries [1, 13, 27–32].

The increasing demand for ophthalmology surgical services, along with increasing backlogs, has brought with it increased waiting times. In Australia in 2016, ophthalmology patients recorded the longest median wait time for elective surgery at 78 days [33], and the average time from referral to first ophthalmology appointment was 188 days [34]. Fast forward to 2021 and the average ophthalmology wait time for elective surgery rose to 118 days [27], extending to 30 months for some cataract surgeries [35]. Survey data from the UK reflects similar trends; according to a poll of 498 Macular Society members, 57% of macular patients experienced a delay whilst waiting for an NHS appointment and/or treatment between 2021 and 2023 [36]. A survey of 557 patients regarding their experiences of eye care services in the last three years revealed that >50% experienced delays, >20% experienced ≥1 appointment or treatment cancellation, and 15% experienced both. 77% felt it had caused them anxiety or stress, whereas 54% felt it had a negative impact on their day-to-day life [37].

Publicly available data on ophthalmic surgical waiting times in Germany, India, and Singapore is limited or unavailable, making it difficult to assess delays and advise targeted policy interventions. This underscores the need for improved data collection and reporting to support evidence-based decision-making and ensure timely, equitable access to ophthalmology services across all regions.

#### Further implications of limited access to care

Limited access to healthcare professionals not only restricts treatment availability but also significantly impacts patient education and support. As a result, patients may be unaware of their surgical or procedural treatment options, which can lead to delayed interventions and worsening outcomes. For instance, a report from the Macular Society in the UK highlights that patients with DME often experience a lack of clear communication about their condition. This contributes to confusion and distress, ultimately affecting adherence to treatment and negatively impacting disease progression [38].

#### Patients awaiting elective surgeries are routinely delayed due to the clinical need of acute surgeries

While underreported in the literature, insights gathered from qualitative clinician interviews across multiple countries highlighted, with varying severity, frequent delays in elective surgeries due to the prioritisation of acute cases. While accommodating urgent cases is a necessary and routine part of healthcare systems to ensure that patients with immediate needs receive timely care, without formal protocols, this practice can lead to rescheduling inefficiencies.

Australian clinicians described how emergency cases dominate daily operating lists, with elective surgeries deliberately under-booked. In the UK, an estimated 25% of elective surgeries are postponed at least once, with no formal protocol for managing rescheduling and handled on a surgeon-by-surgeon basis. In Germany, clinicians offered contrasting views: one reported rare postponement due to structured protocols, while another noting weekly delays of elective surgeries being standard practice. Conversely, in Singapore, elective surgeries are minimally affected, particularly in private settings where dedicated operating theatres for emergencies are maintained. These varying insights highlight the systemic differences in providing timely surgical care across different healthcare models.

#### Summary

The rising global demand for ophthalmology services has led to increasing surgical backlogs and longer wait times, exacerbated by the COVID-19 pandemic. Limited publicly available published data in Germany, India, and Singapore highlights the need for better data transparency. Elective surgeries are delayed due to the prioritisation of acute cases, with varying impacts across countries, highlighting inefficiencies in scheduling and resource management.

Surgical capacity constraints have led to compromised quality of care and poorer patient outcomes.

#### Delayed care leading to preventable vision loss

Surgical CC have led to compromised quality of care and poorer patient outcomes, with the UK serving as a stark example of these challenges. In 2019, ~20 people per month lost their vision due to delays in receiving follow-up care [39], reinforced by 551 NHS clinician reports (2019–2023) of patients in England who lost their sight because of delayed appointments. Of these, 219 cases were classified as “moderate or severe harm,” indicating significant vision impairment, though it is not specified whether the sight loss was temporary or permanent [36]. Moreover, an estimated 235,000 necessary eye surgeries were delayed or missed during the COVID-19 pandemic, equivalent to ~3.5 per 1000 population, with an estimated 2986 people having lost vision due to delayed identification and treatment of eye disease [13]. This is accompanied by a variety of published case reports highlighting the human impact of these delays, illustrating the profound consequences for affected individuals [12, 14, 36]. A recent poll of 498 members of the public with macular eye conditions who required medical intervention in the past two years highlighted the extent of the issue. Nearly six in ten respondents reported experiencing delays while waiting for NHS appointments or treatment. Alarmingly, 47% of respondents had experienced a loss or decline in vision during this time, 21% struggled with day-to-day tasks, 41% expressed fear of losing their vision entirely, while 30% felt abandoned by the NHS or authorities [36]. Ultimately, operational changes, reinforced by data-driven approaches are required to address these challenges. The All-Party Parliamentary Group review of ophthalmology services (2018) emphasised the need for routine data collection to help clinicians prioritise patients most at risk of avoidable sight loss [37], a recommendation that could apply globally due to a lack of robust data regarding waiting times.

#### Delayed care leading to increased falls and associated injuries

Beyond preventable vision loss, delays in essential eye surgeries contribute to increased physical injuries and mental health burdens [40–42]. In Australia, one study reported that ~30% of individuals awaiting cataract surgery experience falls, half resulting in injuries, averaging 1.2 falls and 0.6 injurious falls per person, underscoring the broader health implications of surgical delays [15]. Such delays not only heighten the risk of falls but also exacerbate mental health issues. Untreated cataracts are associated with increased depression and anxiety, while cancelled hospital appointments further contribute to stress in glaucoma patients [42, 43].

#### Summary

Surgical CC have led to compromised quality of care and poorer patient outcomes, with delays causing preventable vision loss, patient harm, and reduced safety across ophthalmology services.

Countries report a shortage of surgical staff (surgeons, nursing and technical staff), exacerbating waiting lists and compounded by growing challenges in recruiting and retaining personnel.

### Workforce challenges

The growing demand for ophthalmology services has placed significant strain on workforces across various healthcare systems, with shortages of surgical staff, including surgeons, nursing staff, and technical personnel, contributing to longer waiting lists and increased workload.

#### Ophthalmologist shortages and projected increases in demand

The UK has 49 ophthalmologists per million people, significantly fewer than Germany’s 81 per million, despite similar economic development, Table 1 [44]. It should be noted publicly available workforce data reports headcount rather than full-time equivalent (FTE). While headcount provides a useful indicator of workforce size, it may overestimate effective capacity where part-time or dual-sector employment is common. Between 2015 and 2020, UK ophthalmology outpatient attendance rose by 12%, while the ophthalmology training workforce grew by only 2.3% [11]. Following this, the RCOphth 2022 workforce census revealed that 76% of NHS ophthalmology units lack sufficient ophthalmology doctors to meet patient demand, with 84% of eye units requiring at least one additional ophthalmologist. However, only 40% of units were actively advertising for these roles [11]. The situation is set to worsen as 26% of ophthalmology doctors plan to leave the workforce within five years, most through retirement, while a further 28% intend to move to independent sector providers [45].

This is compounded by difficulties in recruitment. The 2018 RCOphth Workforce Census reported that 64% of UK eye units struggled to recruit ophthalmology doctors, rising from 58% in 2016 [46]. For specialty and staff-grade roles, 85% of units cited recruitment challenges, primarily due to a lack of suitable candidates [46]. By 2022, 52% of units reported recruitment had become harder in the past year, and 67% reported greater difficulty retaining staff [11]. Consequently, reliance on locum staff (temporary healthcare professionals) has grown, with 65% of UK units employing locums in 2021–22, many for periods exceeding 12 months [11].

Australia has one of the lowest per capita number of ophthalmologists among high-income nations [47], at 29 per million [44], with projections indicating a critical shortage within the next decade [48]. Estimates suggest that even with a yearly growth of 3% in the number of qualified ophthalmologists, there will still be an undersupply by 2030 [49]. Singapore is no better at 24 ophthalmologists per million people [44]. With its aging and growing population, it is projected that the number of Singaporeans with eye conditions will almost double by 2040 [4]. Under the current eye care delivery model, the demand for ophthalmologists is expected to rise significantly, with a projected 117% workforce increase required from 2015 to 2040 [4].

In India, workforce shortages are stark, with only 11 ophthalmologists per million [44]. A 2016 study found that only 58% of eye care facilities in 11 major cities reported having a full-time retina specialist on staff [50].

#### Nursing shortages in ophthalmology and their impact on surgical capacity

Workforce pressures extend beyond ophthalmologists. In Australia, there is a nationwide shortfall in skilled theatre nurses [51], with a 2024 national survey of 238 nurses calling for urgent measures to improve training opportunities to attract nurses to eye care [52]. Concurrently, nursing shortages were cited during expert interviews as a key limiting factor across many healthcare systems. Clinicians reported that while theatre space can be available, procedures are often delayed due to a lack of trained nursing staff. Notably, a German clinician of a major ophthalmology hospital reported vacancies for up to 8 nurses that remain unfilled simply due to the lack of interest. These issues were also reported to extend to imaging technicians in the UK.

While data largely focuses on ophthalmologists, nursing shortages may be an even greater challenge, though the full extent is unclear. Further research is needed to quantify the issue and guide policy interventions, ensuring adequate staffing across all ophthalmic roles. A broader workforce strategy may be required, prioritising investment in nursing, imaging, and technical support staff alongside ophthalmologists.

Expert clinician interviews highlighted significant challenges in retaining nursing staff across Singapore, Australia, Germany, and the UK, particularly younger nurses, due to pay erosion and unattractive working conditions. In Singapore, clinicians noted that burnout is a persistent issue, especially among operating theatre nurses. In Australia, public sector retention is heavily impacted by state-based funding constraints, which limit salary growth, training opportunities, and career progression. As a result, nurses often leave the profession altogether or transition to administrative roles or private practice, where pay and working conditions may be more favourable. These themes were consistent with a 2024 national survey of 238 Australian nurses that found 68.6% of participants planned to exit eye care over the next ten years.

#### Surgical staff are required to work longer hours, sometimes including out-of-hours

The growing demand for ophthalmology services has necessitated extended working hours for surgical staff with published data on extended working hours identified for the UK and Australia. In the UK, a 2019 NHS report found that 43 of 44 units (34% response rate) offered additional clinics, theatre time, or extended hours outside of standard job plans to meet demand [39]. In Australia, a 2013 survey reported that 60% of responding organisations (*n* = 30) had staff working extra hours to manage workloads [53]. These measures provide short-term relief but further intensify pressures on staff. Notably, published data rarely report surgical throughput (e.g., the number of theatre lists or sessions delivered per week), even though throughput is a key determinant of how workforce levels translate into patient access. Addressing these evidence gaps by consistently reporting throughput data would better inform workforce planning and policy.

#### Summary

Staff burnout and stress are more prevalent than before and ever increasing.

Surgical workforce shortages, encompassing surgeons, nursing staff, and technicians, are a critical global issue, leading to longer waiting lists and increased workload. Staffing constraints not only limit the availability of services but also increase reliance on locums, extend working hours, and increase staff attrition, further compounding the challenges in providing timely and effective eye care. In addition, limitations in workforce data reporting, particularly the reliance on headcount rather than FTE and the lack of information on surgical throughput, represent important gaps that hinder effective workforce planning and policy development.

Burnout and stress among ophthalmology professionals, while anecdotally recognised, remain underreported in the literature concerning the countries of interest, necessitating further research. To address this gap, a combination of survey findings and insights from expert interviews were utilised to provide a comprehensive perspective.

#### Survey findings on burnout and stress

Published survey data on burnout were only available from the UK; comparable data were not identified for the other study countries. Although not specific to ophthalmology, the 2023 UK Surgical Workforce Census revealed that 61% of respondents identified burnout and stress as the primary challenge in surgery, largely driven by excessive workloads [54]. This sentiment extends to ophthalmology trainees, as highlighted by the GMC’s 2024 National Training Survey, which found that 8% of ophthalmology trainees reported very high levels of burnout in 2024, down from 14% in 2023. However, more than half (58%) of trainees reported working beyond their rostered hours weekly or daily, significantly higher than the all-specialties average of 40% [55].

#### Interview insights on staff mental well-being related to workforce challenges

Expert interviews corroborated these findings, with UK clinicians reporting colleagues leaving the profession, taking extended sick leave, or emigrating due to overwhelming clinical demands. In Germany, absenteeism among nurses is on the rise, reflecting increasing stress and diminished morale, compounded by a lack of recognition. In Singapore, while burnout is less pronounced in ophthalmology, it affects inpatient care staff and operating theatre nurses, with retaining younger nurses being a persistent challenge.

#### Summary

Time and theatre allocation to training new surgeons is limited due to surgical backlogs.

Burnout and stress among ophthalmology professionals are increasingly prevalent, driven by excessive workloads and systemic pressures. While published survey data were available only from the UK, the interviews suggested that similar workforce pressures may also be present in other countries. Therefore, the absence of published data may not indicate that burnout is less relevant elsewhere but instead reflects a lack of systematically collected evidence. Taken together, the findings highlight the need for future research and dedicated surveys to quantify the prevalence and impact of burnout across different healthcare systems.

#### Challenges in accessing surgical training opportunities

In the UK, the ability to allocate time and theatre space for training new surgeons has been significantly constrained by the demands of managing surgical backlogs. While not specific to ophthalmology, the 2023 UK Surgical Workforce Census cited that 56% of all respondents and 61% surgical trainees found access to theatres was a major challenge [54]. According to a recent report, 86% of trainee ophthalmologists stated difficulty in accessing training programs due to limited opportunities [11]. The RCOphth (2022) noted that 53% of ophthalmology trainees had not been given additional opportunities to complete the required number of practical procedures, a figure notably higher than the 43.5% average across all medical specialties [56]. Furthermore, 74% of ophthalmology trainees expressed a desire to perform more surgical procedures, underscoring the unmet need for training opportunities [11]. This limitation not only affects the skill development of trainees but also poses risks to the future capacity of ophthalmology services.

#### Limited availability of advanced surgical education

In India, the shortage of training resources is reflected in the limited opportunities for advanced surgical education. A 2016 cross-sectional study of eye care facilities in 11 cities revealed that 54% of facilities reported their ophthalmologists sought further training in retina care, highlighting significant gaps in access to specialised training programs [50]. Furthermore, our expert interviews revealed a perceived inequality in the standard/level of training across the country, resulting in a workforce of ophthalmologists with clear varying skill levels.

#### Summary

Limited time and theatre allocation for training new surgeons due to surgical backlogs hinders skill development and poses risks to the future capacity of ophthalmology services. Addressing these constraints is essential to ensure a well-trained workforce capable of meeting long-term patient demand. Unlike the UK and India, comparable data were not identified for Germany, Australia, or Singapore. Similar challenges may exist given the CC in these countries, but they have not been systematically reported in the published literature.

### Impacting factors

Limited surgical/clinic space in some markets

#### Impact of infrastructure limitations

The availability of adequate surgical and clinic space, coupled with the shortage of surgical staff, significantly impacts the capacity to deliver timely and effective ophthalmology care across different healthcare systems. Issues relating to infrastructure and surgical space were most clearly identified in the UK and India; comparable data were not available for Germany, Australia, or Singapore. In the UK, a 2019 NHS report highlighted that many providers struggle to meet capacity demands due to both staffing shortages and physical space limitations. Of the 52 providers surveyed, 49 cited lack of departmental space as a critical barrier to delivering care [39]. A national investigation conducted by the Healthcare Safety Investigation Branch in 2018 reinforced these concerns, particularly in the context of glaucoma follow-up delays. The report found that many hospital eye services lacked adequate clinic space and surgical facilities, which contributed to appointment backlogs and delays in treatment delivery. Some Trusts reported that physical space constraints prevented them from expanding services, even when there was funding or staff available [12].

In India, the shortage of eye health infrastructure is a major challenge, particularly in rural areas. Hospitals often operate as part of chains, individual private clinics, or sub-specialties within multispecialty hospitals [6]. However, rural areas face a lack of primary care delivery, limiting access to essential eye care services and referral systems [6]. These systemic issues highlight the uneven distribution of resources and infrastructure, which impedes access to timely ophthalmology services.

#### Impact of shared surgical space and competing procedural demands

Despite recommendations to the contrary, ophthalmic theatre space is often shared with other specialties, further exacerbating capacity challenges [14, 57]. This issue is then further compounded through competition among various ophthalmic procedures for the same surgical space. For example, certain novel sustained-release drug delivery implants for neovascular AMD patients must compete with simple and intermediate vitrectomy procedures, glaucoma surgeries (e.g., shunt implantations), cataract surgeries, and emergency retinal detachment repairs. The RCOphth has highlighted the importance of prioritising surgeries based on urgency and the potential for vision loss [57]. However, this approach can create additional delays and competing demands for non-urgent cases, further straining ophthalmic surgical capacity.

#### Summary

Limited access to surgical and diagnostic equipment in some countries while technology is advancing at pace.

The combination of limited surgical and clinic space and a shortage of surgical staff in some healthcare systems significantly hinders the delivery of timely ophthalmology care. Addressing these challenges, particularly in regions with uneven resource distribution, is essential to improve capacity and ensure equitable access to services.

#### Challenges in accessing equipment

The availability of appropriate surgical equipment and diagnostic technology is critical for delivering effective ophthalmology services. While diagnostic tools are not inherently surgical, they play a crucial role in early disease detection, enabling timely intervention that may reduce the need for surgical procedures. In this way, access to diagnostic and screening equipment can indirectly support surgical capacity by preventing disease progression and reducing the overall surgical burden [58].

In India, a 2016 cross-sectional study examining public and private eye care facilities across 11 cities revealed that 51.6% of facilities required additional laser or surgical equipment to meet patient demand [50]. In Australia, general practitioners can be reluctant to perform DR screenings due to the high cost of retinal cameras, time constraints, insufficient Medicare incentives, and a limited number of patients seeking such screenings [33]. These gaps not only delay essential surgical and diagnostic interventions but also reduce the overall efficiency of eye care services, exacerbating backlogs and increasing patient wait times.

#### The impact of emerging diagnostic technologies on resource demands

Beyond equipment shortages, advances in ophthalmological treatments are introducing innovative solutions, though investment in surgical infrastructure and technology is required. For instance, transformative therapies, such as long-acting implants, cell-based treatments, and gene therapies, are revolutionising care for conditions like AMD and inherited retinal diseases. For example, sustained-release intravitreal implants for neovascular AMD can provide continuous drug delivery over several months, reducing the need for frequent intravitreal injections, easing both patient and capacity burden [59, 60]. However, the implantation of these devices requires advanced surgical expertise and specialised equipment [61].

#### Summary

Limited access to surgical and diagnostic equipment in some healthcare systems, coupled with the rapid pace of technological advancements, poses significant challenges to delivering equitable and high-quality ophthalmology care. Targeted investments in infrastructure, equipment, and training are critical to addressing these disparities and ensuring patients can benefit from transformative, sight-saving innovations.

Geographic access inequalities – accessing surgical care is much harder in rural locations, even in smaller high-income countries like Germany.

Geographic access inequalities significantly impact access to ophthalmology services, with rural areas facing greater challenges compared to urban regions. This issue persists even in smaller high-income countries like the UK and Germany, as well as in middle-income countries such as India and in geographically vast high-income countries like Australia.

#### Maldistribution of ophthalmologists

In Australia, the maldistribution of ophthalmologists is apparent. As of 2019, according to the AIHW, there were 965 practicing ophthalmologists (40 FTE per million population), but the vast majority, 818, were based in major cities (49 FTE per million population). In contrast, only 23 ophthalmologists worked in outer regional centres, amounting to just 13 FTE per million population [62]. This imbalance is further compounded by the fact that 90% of ophthalmologists graduating between 2013 and 2016 chose to settle in urban areas, continuing the trend of regional shortages [51]. Indigenous Australians are disproportionately affected as more than 60% of the Aboriginal and Torres Strait Islander population reside in regions with little or no access to publicly funded outpatient services [51]. This is further compounded by a notable increase in the age-standardised cataract surgery rate for First Nations Australians from 6462 per million in 2013–14 to 8691 per million in 2020–21 [63]. In addition, access to treatment can be uneven: in Australia, for example, provision of anti-VEGF therapy has been described as a “postcode lottery,” with public availability varying across states and disproportionately affecting rural and lower-income communities [64].

#### Practice location

In Germany, ophthalmology practices are predominantly located in larger towns, resulting in higher travel times for rural inhabitants. A 2016 geographic network-analysis reported that the average round-trip duration to see an ophthalmologist for rural German residents was 129.3 min, compared to 99.3 min for a general practitioner. These barriers are particularly concerning for individuals with poorer socioeconomic status, who are both less likely to participate in screening programs and more likely to need them due to higher health risks [23].

In India, the geographic disparity is stark, with 65% of the population residing in rural areas, while 80% of healthcare workers are based in urban regions [6]. Most major hospitals are located in urban centres [6], leaving rural populations with limited access to eye care. However, outreach initiatives such as the Aravind Eye Care System’s camps have sought to bridge this gap. Between 2018 and 2019, these camps examined 563,941 outpatients (0.41 per 1000 population), performed 88,380 eye surgeries (0.06 per 1000 population), and dispensed over 80,000 pairs of eyeglasses (0.06 per 1000 population), showcasing a model that could be upscaled to address disparities [25].

#### Summary

Geographic disparities make accessing ophthalmology care significantly harder in rural areas, where limited availability of specialists, longer travel times, and fewer resources create barriers to timely care. Targeted interventions, such as outreach programs and improved distribution of healthcare resources, are required in affected areas to reduce inequities and meet the needs of underserved populations.

Shifting reliance on the private sector may prolong public sector waiting times, impacting equitable access to ophthalmic surgery.

#### Increasing reliance on the private sector in some countries

The growing role of the private sector in ophthalmology services has changed how care is delivered, particularly in high-demand procedures like cataract surgery. In the UK, the public-to-private service ratio shifted from 89–11% in 2016 to 54–46% in 2021 [58]. While this increased capacity has helped address surgical volumes, there is no evidence that it has reduced overall waiting times for eye care services [22]. Although many ISP-performed procedures remain NHS-funded, the shift towards private provision risks destabilising NHS eye care units by redistributing workforce and funding, reducing their ability to provide comprehensive services [22]. In the long term, this may extend public-sector waiting times but also raises financial inefficiencies, as outsourcing is often more costly per procedure, diverting resources from sustainable NHS capacity expansion.

#### Dominance of the private sector in some countries

In Singapore, the public-private distribution of ophthalmologists is comparatively more pronounced, with 67% working in the public sector and 33% in private practices as of 2023 [65]. Ophthalmic surgery waiting times differ significantly between the two sectors as private patients can access care much faster, highlighting ongoing inequities in service delivery [66]. In Australia, the imbalance is even greater, with only 16% of ophthalmologists (13% FTE or ~0.5 per 1000 population) working in the public sector as of 2023 due to limited public positions [51]. Of the 250,000 cataract surgeries performed annually in Australia (~9.4 per 1000 population per year), only 28% are conducted in public hospitals [51]. As a result, access to care is heavily influenced by private health insurers, who dictate treatment eligibility, hospital funding, and even prosthetic options, where cost-efficiency can be prioritised over patient need. Meanwhile, although Australia’s Medicare system provides free or low-cost public healthcare, the stark workforce disparity between public and private sectors has led to significant inequality in access: private patients can typically receive surgery within weeks, while public patients often wait over a year [33].

#### Summary

This imbalance between public and private sectors illustrates systemic challenges in providing equitable access to ophthalmology services. Addressing these disparities requires better integration of public and private resources to ensure that patients across all settings can receive timely and effective care.

Increasing workforce capacity to reduce treatment delays by addressing patient demand.

### Solutions & implementations

#### Addressing national and regional shortages

Expanding the ophthalmology workforce is critical to managing increasing patient demand and reducing surgical backlogs. In the UK, 1500 additional undergraduate medical school places were introduced in 2017–18, alongside 15 new national ophthalmology training posts in 2022–23 [67], showing an effort to address shortcomings.

In Australia, the Regionally Enhanced Training Network (RETN) exposes trainees to regional settings, addressing shortages and improving access to care for Aboriginal and Torres Strait Islander populations [68]. Similarly, workforce expansion initiatives in India, such as the Mission Saksham Initiative (2019–2025), aim to train 2000 allied ophthalmic personnel from low socio-economic backgrounds by 2025, increasing service provision in underserved areas. By 2021, 236 students were enrolled in ophthalmic courses, and 24,600 patients received care from graduates of the program, illustrating its impact on workforce expansion and service provision [69].

#### Restructuring ophthalmic care delivery

Upskilling primary care doctors to manage routine ophthalmic cases, expanding the role of optometrists and ophthalmic nurses in public healthcare, and integrating non-medical staff into care pathways can free up ophthalmic surgeons to focus on complex surgical cases [70]. However, significant barriers remain, such as competing manpower demands, regulatory and licensing challenges, lack of data connectivity, and the need for public re-education on these changes [70].

Optimising resources, patient transfer, and appointment management, including triage and digital reminders, to streamline treatment and prioritise urgent cases.

#### Optimising resources and appointment management

Appointment scheduling plays a key role in optimising ophthalmology service delivery. Interviews with German clinicians indicated that biweekly planning meetings, involving representatives from each subspecialty, OR management, and list-assist software, improve slot allocation and manage overbookings. More advanced techniques, such as Discrete Event Simulation, have been applied at the University Eye Hospital of the Ludwig-Maximilian University in 2017 to assess operational solutions before implementation through optimising appointment schedules. Patient waiting times were reduced from 229 ± 100 min to 183 ± 89 min without requiring additional staff [71]. While this study focused on improving general clinic throughput rather than surgical efficiency, similar modelling approaches could help identify strategies for optimising surgical scheduling and resource allocation.

In Singapore, workflow improvements in outpatient eye clinics between 2010 and 2013, such as re-arranging new and follow-up appointments and introducing dilation-free exams, reduced average consultation waiting times from 32 min to 18 min when fully implemented [72].

Some institutions have instead adopted short term but high effort/volume solutions. Milton Keynes Hospital launched the ‘high volume low complexity’ cataract surgery list in 2023, enabling more patients to be treated over shorter timeframes by streamlining resources, carefully prioritising cases, improving patient transfer processes, and optimising high-level organisation [73].

#### Digital reminders and patient education

A report by Macular Disease Foundation Australia found that increasing treatment persistence in just 25% of patients currently receiving Anti-VEGF intravitreal injections could lead to an additional 22,000 people continuing necessary treatment who would otherwise discontinue therapy, highlighting the importance of patient education and digital reminders [64]. By improving long-term disease management, such strategies may reduce the number of patients progressing to advanced stages that require surgical intervention, ultimately easing pressure on surgical capacity.

Artificial intelligence to optimise scheduling, patient flow, and early screening processes to improve efficiency.

AI-driven technologies are revolutionising ophthalmology by enhancing screening accuracy, patient triage, and workflow optimisation, leading to improved efficiency and resource allocation. These advancements have significant implications for surgical capacity by reducing unnecessary referrals, prioritising urgent cases, and streamlining patient flow to ensure timely surgical intervention for those who need it most.

#### AI-enhanced screening and disease detection

Several studies have demonstrated AI’s role in early detection and triage, which can reduce surgical burden by preventing disease progression or ensuring that surgical cases are identified and scheduled more efficiently.

- Semi-Automated DR Screening Model (Singapore, 2015) – By automating fundus photography triage, this system has reduced reliance on specialists for early-stage assessments, freeing up surgical ophthalmologists for more complex cases. This approach has saved the healthcare system in Singapore an estimated $489,000 annually, with projected savings of $15 million by 2050, while freeing up eye care personnel for other tasks [74].
- Singapore Eye Lesion Analyzer (SELENA, 2019) – Enables early detection of DR, AMD, and glaucoma, allowing for earlier procedural or surgical intervention where needed, preventing further deterioration and reducing late-stage surgical backlogs. Particularly recommended for regions with limited ophthalmologists [75].
- Eye2Gene algorithm - AI-driven genetic screening tool for inherited retinal diseases, achieving 85.6% accuracy in identifying the genetic cause within its top five diagnoses, expanding global access to specialist expertise [76].
- Moorfields Eye Hospital AI Study (UK) – Advanced AI models compare new retina scans to historical databases, accurately identifying over 50 eye diseases in 94% of cases [39].

#### AI-driven patient scheduling and workflow optimisation

AI is increasingly being used to enhance patient scheduling and operating theatre efficiency, ensuring that surgical slots are allocated effectively, and that no-shows or overbookings are minimised [77–81]. Of note, the DemDx Ophthalmology Triage System (DOTS), was developed to optimise triage in ophthalmic emergency departments, ensuring that patients are prioritised appropriately. Using retrospective data from 11,315 attendances at MEH in London, DOTS classified cases as “see immediately,” “see within a week,” or “see electively.” It was validated temporally, compared to triage nurses, and externally tested at a Brazilian hospital. DOTS achieved high sensitivity (94.5% at MEH, 95.2% externally) and better specificity (42.4% at MEH, 32.2% externally) compared to nurses (25.1% specificity). Its performance remained robust across varying disease incidences and socio-demographic subgroups.

Mobile clinics/outreach services to expand surgical capacity and increase access to care by providing additional space and services, particularly in rural areas.

Expanding access to ophthalmology services through mobile clinics and outreach programs has proven effective, particularly in rural areas. In India, non-governmental organisations such as the LV Prasad Eye Institute and Aravind Eye Hospitals have worked to address geographic disparities. They support rural populations through a cross-subsidisation model, offering complimentary eye services and achieving cataract surgery success rates of 97%, far above the national average of 73% [82]. In the UK, University Hospitals Plymouth NHS Trust, supported by the NHS Elective Recovery Fund, deployed two mobile operating theatres to relocate existing ophthalmic services to purpose-built units, thereby increasing surgical capacity [83]. Similarly, in Australia, outreach initiatives like Lions Outback Vision and the Griffith Base Hospital Eye Care Model (2022–2023) significantly reduced waitlists. The latter cut the number of patients from 123 to just 10 within a year by bringing Sydney-based ophthalmologists to regional facilities [84]. Prior to the commencement of construction, communities and patient groups should be consulted on the proposed locations for new facilitates or mobile clinics, so that decisions allow for community engagement.

Telemedicine to enable remote monitoring, reserving in-person resources for complex cases needing direct assessment.

#### Successful integration in clinics globally

Telemedicine initiatives have enhanced efficiency by reducing unnecessary in-person visits, improving remote monitoring, and streamlining preoperative assessments, allowing surgical resources to be allocated more effectively.

- Singapore Integrated Diabetic Retinopathy Program (SiDRP) (2016) – This model processed over 150,000 screenings across 20 centres achieving 90% sensitivity and specificity while significantly reducing diagnostic turnaround times [85, 86].
- Germany’s Collaborative Telemedicine Programs – Enabled optometrists to perform routine diagnostic assessments (e.g., OCT-A), freeing ophthalmologists to focus on procedural and surgical cases [87, 88].

#### At home monitoring solutions

Emerging at-home monitoring solutions are further advancing the capabilities of telemedicine by empowering patients to monitor their vision remotely, reducing avoidable in-person follow-ups and allowing earlier detection of cases requiring intervention.

Earlier utilisation of more effective, longer acting, advance treatments capable of being delivered by a range of HCPs, to effectively lower patient and health system burden.

The adoption of longer-acting, advanced treatments in ophthalmology represents a pivotal shift in reducing the strain on healthcare systems and improving patient outcomes by optimising resource allocation and care delivery. By extending treatment intervals and reducing the need for frequent in-person procedures, these therapies help prevent disease progression, lowering the number of patients who require surgical intervention. However, successful integration of these therapies requires a sufficient reserve to support their adoption. While longer-acting treatments reduce resource burden, their implementation depends on financial investment, clinician training, and workflow adjustments. Without adequate support, adoption may be slow, limiting their impact on CC and service efficiency.

## Conclusions

The growing CC in surgical ophthalmology demands urgent policy action to prevent worsening delays and inequities in patient care. Across healthcare systems, common challenges include surgical workforce shortages, infrastructure limitations, inefficiencies in scheduling and resource allocation, and an increasing reliance on private sector providers. Many countries also face geographic disparities, where rural and underserved populations experience significantly reduced access to timely ophthalmic care. While these issues are widespread, their impact varies by healthcare system, requiring tailored policy responses that reflect local workforce capacity, funding models, and service structures.

This review was deliberately broad in scope, spanning multiple countries and intervention types, to highlight common and system-wide capacity challenges rather than provide an in-depth analysis of a single intervention. By framing the evidence in this way, our intention was to surface cross-cutting themes and identify transferable solutions relevant to policymakers. This review serves as a call-to-action for policymakers, healthcare leaders, and industry stakeholders to implement coordinated, evidence-based reforms adapted to the specific needs of each healthcare system. Bold, collaborative efforts to invest in and implement sustainable, scalable solutions, such as workforce expansion, telemedicine, AI-assisted diagnostics, and mobile clinics, are required. These proven interventions can alleviate backlogs, optimise resources, and improve access to care, particularly in underserved regions. Our findings reinforce the conclusions of recent work in the field, together pointing to an urgent need for coordinated policy action in ophthalmology [89]. The time for meaningful, country-specific reform is now to ensure healthcare systems can meet future demand and provide equitable, timely surgical care.

## Supplementary information

Supplemental material

## Data Availability

All data generated or analysed during this study are included in this published article and its supplementary information files.
