# Supplementary material for: Surgical capacity in ophthalmology: the unmet need for sustainable solutions
Source: Eye (Lond). 2025 Dec 16;40(4):452–64. doi: 10.1038/s41433-025-04153-x (PMC12957439; doi:10.1038/s41433-025-04153-x)
Supplement: Supplementary file 1 — Supplemental material [file 41433_2025_4153_MOESM1_ESM.docx]

**Supplementary Information**

Details of the PICOS (Population, Intervention, Comparator, Outcomes, Study design) criteria used to guide study selection during the targeted literature review.

| Supplementary Table 1. PICOS requirements for the targeted literature review. | |
| --- | --- |
| PICOS elements | |
| Population | - General ophthalmology - AMD, DMO/DME, DR, GA, cataract surgery |
| Intervention/  Comparator | - No criteria/limitations |
| Outcomes | Data pertaining to:   - Disease prevalence - Waiting lists and waiting times - Workforce - Clinic space - Clinical impact of capacity constraints - Economic impact of capacity constraints - Impact of COVID-19 - Solutions |
| Study types | - No limitations |
| Date range | - Studies published in the last 10 years |
| Country focus | - UK, Australia, Germany, Singapore, India |
| Language | - English-language articles only. Government websites and some non-English articles were translated where feasible, but non-English sources were not comprehensively searched. |
| Abbreviations: AMD, age-related macular degeneration; DME, diabetic macular oedema; DMO, diabetic macular oedema; DR, diabetic retinopathy; GA, geographic atrophy; UK, United Kingdom. | |

| Supplementary Table 2. Interview discussion guide | |
| --- | --- |
| General perspective of capacity constraints | |
| 1. | What is your overall perception of the current capacity constraints facing the field of ophthalmology, particularly how they affect general ophthalmology services and how they compare to surgical specialties? |
| 1a. | Could you detail specific capacity constraints faced by surgical ophthalmology in [UK/Germany/Australia/Singapore/India]? |
| 1b. | What is the total capacity in terms of patients seen and/or treated per annum in your market and clinic? |
| Capacity constraints in your clinic | |
| 2. | How would you rate the overall operational capacity of the ophthalmology department as a whole at your clinic (scale 1-10)? Please provide rationale for your rating. |
| 3. | Using the same scale, how would you rate the surgical capacity within your ophthalmology clinic? What are the specific challenges or factors that influence this rating? |
| 4. | Could you provide an estimate of the typical waiting time for patients requiring surgical ophthalmology procedures in your clinic? How does this compare specifically with back-of-eye surgeries? |
| 4a. | Regarding the waiting lists for these surgeries, how long are they currently? Prompts: number of patients |
| 4ai. | Have there been any changes or trends in the length of these waiting lists over the past year or past few years? |
| 5. | Do you currently have sufficient numbers of surgical staff (including surgeons and support personnel) to meet patient demand? |
| 6. | Given that the prevalence of retinal diseases and other conditions requiring surgery has risen in recent years and is projected to rise further, to what extent is your clinic prepared for this increase in patient numbers from a surgical perspective? Please provide rationale for your rating. |
| 6a. | Is anyone at regional or national level responsible for forecasting this increase? |
| 6ai. | If yes, how are they planning to manage this anticipated growth? |
| 6aii. | If no, ask if there is a responsible person at their clinic |
| 7. | In your opinion, are sufficient ophthalmology surgeons being trained to meet current and future demands? |
| 7a. | If not, what is the cause and how could it be improved? |
| 7b. | Is there a difference in the training pathways between general ophthalmologists and ophthalmology surgeons (in route or capacity)? |
| 8. | To what extent does the availability of surgical and clinic space contribute to capacity constraints in your clinic? |
| 8a. | How have recent (and potential future) advancements in technologies, which might require dedicated space and/or more staff time per patient, affected capacity issues in your clinic? |
| 9. | Based on your experience, how have surgical capacity constraints impacted clinical outcomes for patients? |
| 10. | How have surgical capacity constraints impacted the operational aspects of your surgical ophthalmology services? |
| 11. | When facing capacity constraints, acute cases may take precedence over elective surgeries, leading to postponements. To what extent are elective surgeries not performed or postponed due to the prioritization of acute cases in your clinic? |
| 12. | How does your clinic manage the scheduling challenges between elective and acute surgeries? |
| 12a. | a. Are there specific protocols for handling multiple postponements of elective surgeries? |
| Geographic disparities in accessing care | |
| 13. | Are you aware of any geographical disparities (in your country or region) in accessing surgical care or interventions? |
| 13a. | How is surgical care distributed over the country/region?  Probe: Are surgical interventions centralised or available at all ophthalmology clinics? How does this impact the patient? |
| Solutions | |
| 14. | Have any policies or strategies been implemented in your clinic to specifically address surgical capacity issues? |
| 14a. | What were these measures, and how effective have they been in mitigating the problems? |
| 15. | Are you aware of any strategies employed in other regions or countries that effectively address surgical capacity constraints? |
| Close out | |
| 16. | Are there any other key elements impacting surgical capacity constraints in ophthalmology in your experience that we have not already discussed? |
